# Pulmonary haemorrhage and haemoptysis associated with bevacizumab-related treatment regimens: a retrospective, pharmacovigilance study using the FAERS database

**DOI:** 10.3389/fphar.2024.1339505

**Published:** 2024-06-24

**Authors:** Huiping Hu, Zhiwen Fu, Jinmei Liu, Cong Zhang, Shijun Li, Yu Zhang, Ruxu You

**Affiliations:** ^1^ Department of Pharmacy, Union Hospital, Tongji Medical College, Huazhong University of Science and Technology, Wuhan, China; ^2^ Department of Pharmacy Administration and Clinical Pharmacy, School of Pharmaceutical Sciences, Peking University, Beijing, China

**Keywords:** bevacizumab, pulmonary haemorrhage, haemoptysis, pharmacovigilance analysis, FAERS

## Abstract

**Background:** Bevacizumab (BV) is widely used in routine cancer treatment and clinical therapy in combination with many other agents. This study aims to describe and analyse post-market cases of pulmonary haemorrhage and haemoptysis reported with different BV treatment regimens by mining data from the United States Food and Drug Administration Adverse Event Reporting System (FAERS) database.

**Methods:** Data were collected from the FAERS database between 2004 Q1 and 2023 Q1. Disproportionality analysis including the reporting odds ratio (ROR) was employed to quantify the signals of disproportionate reporting of pulmonary haemorrhage and haemoptysis adverse events (AEs) associated with BV-related treatment regimens. The demographic characteristics, time to onset and outcomes were further clarified.

**Results:** A total of 55,184 BV-associated reports were extracted from the FAERS database, of which 497 reports related to pulmonary haemorrhage and haemoptysis. Overall, the median onset time of pulmonary haemorrhage and haemoptysis AEs was 43 days (interquartile range (IQR) 15-117 days). In the subgroup analysis, BV plus targeted therapy had the longest median onset time of 90.5 days (IQR 34-178.5 days), while BV plus chemotherapy had the shortest of 40.5 days (IQR 14–90.25). BV plus chemotherapy disproportionately reported the highest percentage of death (148 deaths out of 292 cases, 50.68%). Moreover, the BV-related treatments including four subgroups in our study demonstrated the positive signals with the association of disproportionate reporting of pulmonary haemorrhage and haemoptysis. Notably, BV plus chemotherapy showed a significant higher reporting risk in pulmonary haemorrhage and haemoptysis signals of disproportionate reporting in comparison to BV monotherapy (ROR 5.35 [95% CI, 4.78–6.02] vs. ROR 4.19 [95% CI, 3.56–4.91], *p* = 0.0147).

**Conclusion:** This study characterized the reporting of pulmonary haemorrhage and haemoptysis, along with the time to onset and demographic characteristics among different BV-related treatment options. It could provide valuable evidence for further studies and clinical practice of BV.

## 1 Introduction

Bevacizumab (BV), a recombinant humanized monoclonal antibody against vascular endothelial growth factor (VEGF), inhibits tumour growth by blocking angiogenesis (Kanbayashi et al., 2022). By specifically binding to VEGF ligand, BV inhibits VEGF ligand-receptor binding and thereby prevents new vessel formation, regresses existing vessels and normalizes tumour vessel permeability (Garcia et al., 2020). BV was first approved for metastatic colorectal cancer (CRC) by the United States Food and Drug Administration (FDA), and then extended to its application for a variety of advanced solid tumors, including non-small cell lung cancer (NSCLC), glioblastoma, metastatic renal cell cancer (RCC), advanced cervical cancer, epithelial ovarian cancer, fallopian tube cancer, primary peritoneal cancer and hepatocellular carcinoma (HCC) (Ferrara et al., 2004; Bai et al., 2021; Giantonio et al., 2023).

Effective pharmacotherapy with BV requires appropriate management of adverse events (AEs) that may occur with BV treatment. Though BV is a well-tolerated anti-tumor drug with a relative safety profile and manageable AEs, it is worth noting that the side effects of BV are different from those of traditional chemotherapy. In contrast to the common bone marrow suppression and gastrointestinal toxicity with chemotherapy, AEs reported with BV include hypertension, hemorrhage, proteinuria, and gastrointestinal perforation (Hatake et al., 2016; Motoo et al., 2019; Kanbayashi et al., 2020). The importance of BV-associated hemorrhage is highlighted by a warning issued by the FDA which recognizes that severe or fatal hemorrhage, including haemoptysis (the spitting of blood derived from the lungs or bronchial tubes as a result of pulmonary hemorrhage), gastrointestinal bleeding, hematemesis, central nervous system (CNS) hemorrhage, epistaxis, and vaginal bleeding, occurred up to 5-fold more frequently in patients receiving BV compared to patients receiving chemotherapy alone (Shimoyama et al., 2009). Serious or fatal pulmonary haemorrhage occurred in 31% of patients with squamous NSCLC and 4% of patients with non-squamous NSCLC receiving BV with chemotherapy compared to none of the patients receiving chemotherapy alone (Garcia et al., 2020). Hemorrhage events such as pulmonary haemorrhage and haemoptysis, represent some of the most severe AEs associated with BV therapy in clinical trials, with certain cases resulting in fatalities (Reck et al., 2009; Dansin et al., 2012; Allegra et al., 2013; Bennouna et al., 2013; Cunningham et al., 2013; Johnson et al., 2023).

Despite the severity of BV-induced pulmonary haemorrhage and haemoptysis, there have been few descriptive studies to characterise these AEs, lacking detailed AE information. The risk of pulmonary haemorrhage and haemoptysis events during the different treatments with BV in cancer patients has also not been elucidated clearly. In addition, due to the intricate biological interactions inherent in BV combination therapies, the emergence of new AEs and the exacerbation of existing ones are possible (Gu et al., 2023), current research on the comparison of pulmonary haemorrhage and haemoptysis between different combination treatments related to BV is extremely limited. In addition, the systematic study on pulmonary haemorrhage and haemoptysis event signals of disproportionate reporting related to BV-related treatment regimens based on large international and real-world databases remains still insufficient.

Spontaneous reporting system (SRS) has become an important information source for exploring post-marketing drug safety with the characteristics of a wide monitoring range and earlier detection of suspected AE signals of disproportionate reporting (Gu et al., 2023). The United States Food and Drug Administration Adverse Event Reporting System (FAERS) is a public and accessible database designed to support the FDA’s post-marketing safety monitoring of drugs and therapeutic biologic products. Previously unknown potential drug-AE associations and well-established clinical associations can be identified by mining the FAERS database.

Herein, we performed a retrospective pharmacovigilance study to investigate the pulmonary haemorrhage and haemoptysis reported in association with BV-related therapies and examine the difference between pulmonary haemorrhage and haemoptysis events and different BV-related treatment regimens (including BV monotherapy, BV plus chemotherapy, BV plus ICI and BV plus targeted therapy) based on the FAERS (Oshima et al., 2018; Salem et al., 2018; Zhu et al., 2021). We identified the pulmonary haemorrhage and haemoptysis AEs signals of disproportionate reporting using the reporting odds ratio (ROR), and further clarified the demographic characteristics, time to onset and outcomes.

## 2 Materials and methods

### 2.1 Data sources

This retrospective pharmacovigilance study utilized data from the FDA adverse event reporting system (FAERS) database (https://fis.fda.gov/extensions/FPD-QDE-FAERS/FPD-QDE-FAERS.html). FAERS database is a publicly available post-marketing database for the safety surveillance of a drug, which collects adverse events (AEs) reported by consumers, health professionals and others. It contains seven datasets, including demographic and administrative information (DEMO), drug information (DRUG), indications of drugs (INDI), outcome information (OUTC), adverse drug reaction information (REAC), report sources (RPSR), therapy start and end dates of the reported drugs (THER).

### 2.2 Data extraction and cleaning

The FAERS database inevitably includes duplicate data because of the spontaneity of the reports. Therefore, the deduplication process is necessary to minimize both false-negatives and false-positives. According to FDA recommendations, with the same CASEID, the latest FDA_DT is selected, or when the CASEID and FDA_DT were the same, the higher PRIMARYID was selected to remove duplicate records (Poluzzi et al., 2012). In this study, We extracted AE data from the FAERS quarterly data files from the first quarter of 2004 (Q1 2004) to the first quarter of 2023 (Q1 2023) using the search terms “Bevacizumab” and “Avastin” (not including biosimilar forms of bevacizumab). AEs in the FAERS database are coded according to the preferred terms (PTs) derived from the Medical Dictionary for Regulatory Activities (MedDRA) version 26.0. Cases with the preferred term Pulmonary haemorrhage and Haemoptysis were included. Then, according to the medication regimen, these data were divided into the following four categories: BV monotherapy, BV plus chemotherapy, BV plus immune checkpoint inhibitor (ICI), and BV plus targeted therapy. Details for these drug names encompassed within chemotherapy, ICI, and targeted therapy are listed in Table 1.

**TABLE 1:** Summary of chemotherapy, ICIs, and targeted therapy drug names.

Categories	Drug names
Chemotherapy	Platinum drugs: Cisplatin; Carboplatin; Paraplatin; Nedaplatin; Oxaliplatin
	Pemetrexed: Pemetrexed; Alimta
	Gemcitabine: Gemcitabine; Gemzar
	Taxoid drugs: Paclitaxel; Taxol; Albumin-bound paclitaxel; Nab-paclitaxel; Abraxane; Docetaxel; Taxotere; Anzatax
	Vindesine: Vindesine; Vinorelbine; Navelbine
	Etoposide: Etoposide; VP-16
	Other drugs: Irinotecan; Topotecan; Mitomycin; Amrubicin; Ifosfamide; Cyclophosphamide; Bortezomib; Everolimus; Temozolomide; Thalomid; Capecitabine; Fluorouracil; 5-FU
ICIs	Anti-PD-1 inhibitors: Nivolumab; Pembrolizumab; Cemiplimab; Opdivo; Keytruda; Libtayo
	Anti-PD-L1 inhibitors: Atezolizumab; Durvalumab; Avelumab; Imfinzi; Bavencio; Tecentriq
	Anti-CTLA4 inhibitors: Ipilimumab; Tremelimumab; Yervoy
Targeted therapy	EGFR-TKI: Iressa; Gefitinib; Tarceva; Erlotinib; Gilotrif; Afatinib; Tagrisso; Osimertinib; Dacomitinib; Vizimpro; Lapatinib; Tykerb; Icotinib; Conmana
	EGFR antibody: Cetuximab; Erbitux
	ALK-TKI: Crizotinib; Xalkori; Alectinib; Alecensa; Ceritinib; Zykadia; Entrectinib; Rozlytrek; Brigatinib; Alunbrig; Lorlatinib; Lorviqua
	Other drugs: Cediranib; Temsirolimus (CCI-779); Endostatin; Sorafenib; Herceptin; Trastuzumab; Rituxan; Rituximab; Trebananib (AMG 386); Endostatin; Faslodex; Lucentis

### 2.3 Time-to-onset analysis

The onset time of pulmonary haemorrhage and haemoptysis was calculated by subtracting the event start date (EVENT_DT) in the “DEMO” file from the treatment start date (START_DT) in the “THER” file. To ensure the accuracy of calculation, we excluded cases with partial date or without date, and then further excluded cases with input errors (EVENT_DT earlier than START_DT). Cumulative distribution curves were used for the demonstration of time-to-onset across comparison groups.

### 2.4 Descriptive analysis

A comprehensive descriptive analysis was performed to summarize the clinical characteristics of FAERS reports documenting BV-related haemoptysis/pulmonary haemorrhage events. We retrieved and described detailed information, including gender, indication, outcome, reported country and the type of reporter (health professional or others) whenever this data was available. It should be noted that the descriptive analysis of age information was not conducted, because age information was only reported for three cases and missed for the others.

### 2.5 Statistical analysis

Disproportionality analysis, which is a widely used approach in pharmacovigilance study, was used to detect potential AE signals of disproportionate reporting for BV in this studies. The reporting odds ratio (ROR) was used to compare the number of haemoptysis/pulmonary haemorrhage events related to different BV combined treatment strategies to the full database. Calculations of ROR and 95% confidence interval (CI) were based on 2 × 2 contingency table (Zhai et al., 2019; Gu et al., 2023), the 2 × 2 contingency table was shown in Table 2. Specific formulas were shown below:

$$ROR=\frac{\frac{a}{b}}{\frac{c}{d}}=\frac{a*d}{b*c}$$

$$95\%CI=e^{\ln\left(ROR\right)\pm 1.96*\sqrt{\frac{1}{a}+\frac{1}{b}+\frac{1}{c}+\frac{1}{d}}}$$

**TABLE 2:** A 2 × 2 contingency table for disproportionality analysis.

	Pulmonary haemorrhage and haemoptysis AEs	Non-pulmonary haemorrhage and haemoptysis AEs	Total
Drugs of interest (BV-related subgroup)	a	b	a + b
Other drugs	c	d	c + d
Total	a + c	b + d	a + b + c + d

The positive signal of disproportionate reporting was defined when the lower limit of the 95% CI of ROR exceeded one, with at least three cases (Guo et al., 2023). In this study, all data processing and statistical analyses were performed using SAS version 9.4 (SAS Institute Inc., Cary, NC, United States), Microsoft EXCEL 2016 and GraphPad Prism 6.0 (GraphPad Software, CA, United States). A chi-square test was used to compare the differences between subgroups. The result of *p* < 0.05 was considered statistically significant.

## 3 Results

### 3.1 Data preparation

**FIGURE 1:**
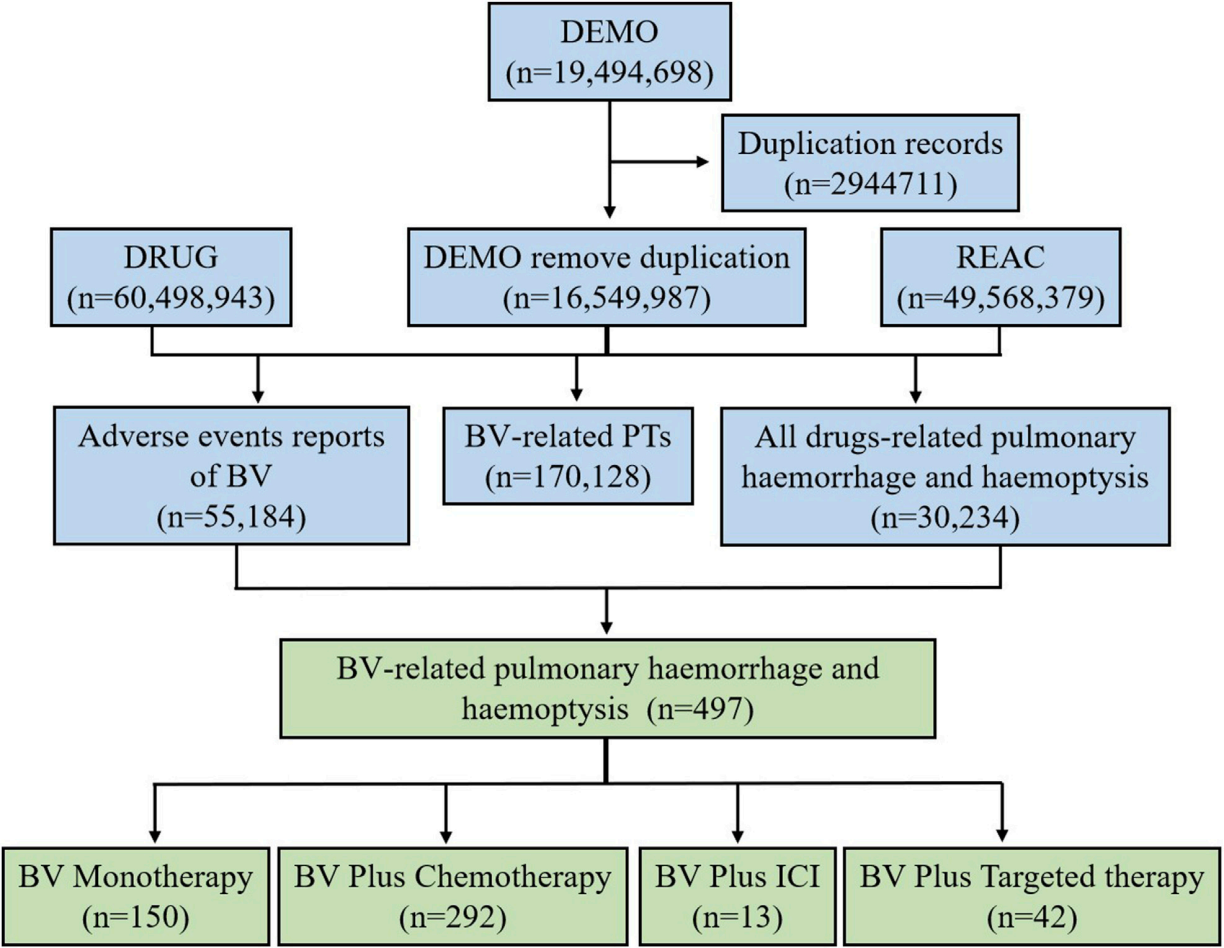
The flow diagram of selecting pulmonary haemorrhage and haemoptysis cases reported in association with BV-related regimens from the FAERS database.

During the period of this study (Q1 2004-Q1 2023), a total of 19,494,698 reports were extracted from the FAERS database. After the deduplication, culminating in the extraction of 16,549,987 unique AE reports. Among these, there were 55,184 AE reports associated with the use of BV. A cumulative total of 30,234 pulmonary haemorrhage and haemoptysis cases that remained in the dataset (for all drugs, drug-event pairs). And there were 170,128 BV-related PTs (drug-event pairs). After processing, we obtained 497 reports of the BV reporting pulmonary haemorrhage and haemoptysis. Then the 497 reports were divided into the following four BV-related subgroups according to the medication regimen: BV monotherapy (n = 150), BV plus chemotherapy (n = 292), BV plus ICI (n = 13), and BV plus targeted therapy (n = 42). The flow diagram of our study is shown in Figure 1.

### 3.2 Demographics description

The demographical characteristics are described in Table 3. The proportion of men was greater than that of women (42.9% vs. 34.6%), this trend was also observed in each subgroup. Most cases were reported in 2014-2018 (35.8%), whereas the BV plus ICI group all reported in 2019-2023, indicating the increased application of ICIs in recent years. According to the data, lung cancer was the most reported indication (53.1%). Death was the most frequently reported serious outcome, accounting for 45.5%. Among them, 148 (50.7%) death cases were reported by the BV plus chemotherapy group, higher than other groups. The United States (48.5%) reported the most pulmonary haemorrhage and haemoptysis AE, followed by Japan (14.3%), China (6.4%), Germany (6.0%), and the United Kingdom (5.4%). In addition, more than half of the reports (58.6%) were submitted by physicians (MD), while other health professionals (OT) were the second largest source of reports, accounting for 18.7%.

**TABLE 3:** Clinical characteristics of pulmonary haemorrhage and haemoptysis cases reported for BV-related regimens from the FAERS database.

Characteristics	Overall (n = 497)	BV monotherapy (n = 150)	BV plus chemotherapy (n = 292)	BV plus ICI (n = 13)	BV plus targeted therapy (n = 42)
Gender					
Female	172 (34.6%)	42 (28.0%)	111 (38.0%)	3 (23.1%)	16 (38.1%)
Male	213 (42.9%)	66 (44.0%)	121 (41.4%)	9 (69.2%)	17 (40.5%)
Unknown	112 (22.5%)	42 (28.0%)	60 (20.5%)	1 (7.7%)	9 (21.4%)
Reporting year					
2019–2023	91 (18.3%)	27 (18.0%)	47 (16.1%)	13 (100.0%)	4 (9.5%)
2014–2018	178 (35.8%)	67 (44.7%)	91 (31.2%)	0 (0.0%)	20 (47.6%)
2009–2013	143 (28.8%)	31 (20.7%)	105 (36.0%)	0 (0.0%)	7 (16.7%)
2008 and before	85 (17.1%)	25 (16.7%)	49 (16.8%)	0 (0.0%)	11 (26.2%)
Indications					
Lung cancer	264 (53.1%)	60 (40.0%)	176 (60.3%)	10 (76.9%)	18 (42.9%)
Colorectal cancer	61 (12.3%)	21 (14.0%)	39 (13.4%)	0 (0.0%)	1 (2.4%)
Breast cancer	35 (7.0%)	7 (4.7%)	24 (8.2%)	0 (0.0%)	4 (9.5%)
Renal cancer	19 (3.8%)	14 (9.3%)	3 (1.0%)	0 (0.0%)	2 (4.8%)
Gastric cancer	9 (1.8%)	0 (0.0%)	4 (1.4%)	0 (0.0%)	5 (11.9%)
Head and neck cancer	8 (1.6%)	2 (1.3%)	5 (1.7%)	0 (0.0%)	1 (2.4%)
Ovarian cancer	7 (1.4%)	2 (1.3%)	4 (1.4%)	0 (0.0%)	1 (2.4%)
Uterus cancer	7 (1.4%)	1 (0.7%)	6 (2.1%)	0 (0.0%)	0 (0.0%)
Liver cancer	6 (1.2%)	2 (1.3%)	1 (0.3%)	3 (23.1%)	0 (0.0%)
Others	27 (5.4%)	12 (8.0%)	9 (3.1%)	0 (0.0%)	6 (14.3%)
Unspecified	54 (10.9%)	29 (19.3%)	21 (7.2%)	0 (0.0%)	4 (9.5%)
Serious outcomes					
Death (DE)	226 (45.5%)	57 (38.0%)	148 (50.7%)	6 (46.2%)	15 (35.7%)
Life-threatening (LT)	8 (1.6%)	1 (0.7%)	6 (2.1%)	1 (7.7%)	0 (0.0%)
Hospitalization–initial or prolonged (HO)	96 (19.3%)	25 (16.7%)	53 (18.2%)	3 (23.1%)	15 (35.7%)
Disability (DS)	3 (0.6%)	0 (0.0%)	3 (1.0%)	0 (0.0%)	0 (0.0%)
Other serious (important medical event) (OT)	121 (24.3%)	48 (32.0%)	59 (20.2%)	2 (15.4%)	12 (28.6%)
Unspecified	43 (8.7%)	19 (12.7%)	23 (7.9%)	1 (7.7%)	0 (0.0%)
Reported countries					
United states	241 (48.5%)	96 (64.0%)	114 (39.0%)	3 (23.1%)	28 (66.7%)
Japan	71 (14.3%)	8 (5.3%)	56 (19.2%)	5 (38.5%)	2 (4.8%)
China	32 (6.4%)	12 (8.0%)	17 (5.8%)	0 (0.0%)	3 (7.1%)
United Kingdom	27 (5.4%)	5 (3.3%)	18 (6.2%)	1 (7.7%)	3 (7.1%)
Germany	30 (6.0%)	8 (5.3%)	19 (6.5%)	0 (0.0%)	3 (7.1%)
Others	79 (15.9%)	17 (11.3%)	55 (18.8%)	4 (30.8%)	3 (7.1%)
Unspecified	17 (3.4%)	4 (2.7%)	13 (4.5%)	0 (0.0%)	0 (0.0%)
Reporters					
Physicians (MD)	291 (58.6%)	77 (51.3%)	178 (61.0%)	10 (76.9%)	26 (61.9%)
Pharmacist (PH)	27 (5.4%)	15 (10.0%)	10 (3.4%)	0 (0.0%)	2 (4.8%)
Consumer (CN)	46 (9.3%)	25 (16.7%)	19 (6.5%)	1 (7.7%)	1 (2.4%)
health professional (HP)	25 (5.0%)	3 (2.0%)	18 (6.2%)	2 (15.4%)	2 (4.8%)
Other health professional (OT)	98 (18.7%)	25 (16.7%)	58 (19.9%)	0 (0.0%)	10 (23.8%)
Unspecified	15 (3.0%)	5 (3.3%)	9 (3.1%)	0 (0.0%)	1 (2.4%)

### 3.3 Time to event onset

**FIGURE 2:**
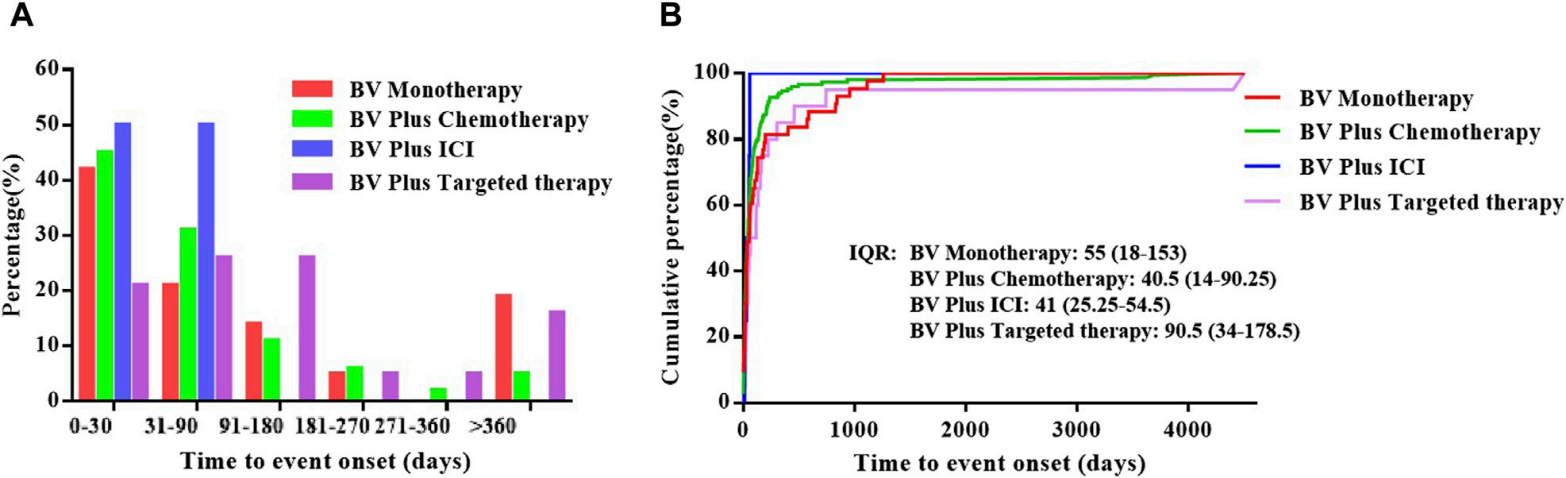
The time to onset of pulmonary haemorrhage and haemoptysis cases reported for BV-related regimens in different subgroups. **(A)** The percentage of the onset time of pulmonary haemorrhage and haemoptysis cases reported in association with BV-related regimens, **(B)** The cumulative distribution curve of time to event onset.

After data cleaning, a total of 217 records were used for time-to-onset analysis, with 43 records in the BV monotherapy, 150 records in the BV plus chemotherapy, 4 records in the BV plus ICI and 20 records in the BV plus targeted therapy. The onset time of pulmonary haemorrhage and haemoptysis for each BV-related regimen is shown in Figure 2A and Supplementary Table S1. Overall, the median onset time of pulmonary haemorrhage and haemoptysis AEs was 43 days (interquartile range (IQR) 15-117 days) after all BV-related categories initiation. As shown in Figure 2B and Supplementary Table S2, the longest median onset time was 90.5 (IQR 34–178.5) days for BV plus targeted therapy, while the shortest of 40.5 (IQR 14–90.25) days for BV plus chemotherapy, and 41 (IQR 25.25–54.5) days for plus ICI, 55 (IQR 18–153) days for BV monotherapy, respectively.

### 3.4 Outcome

**FIGURE 3:**
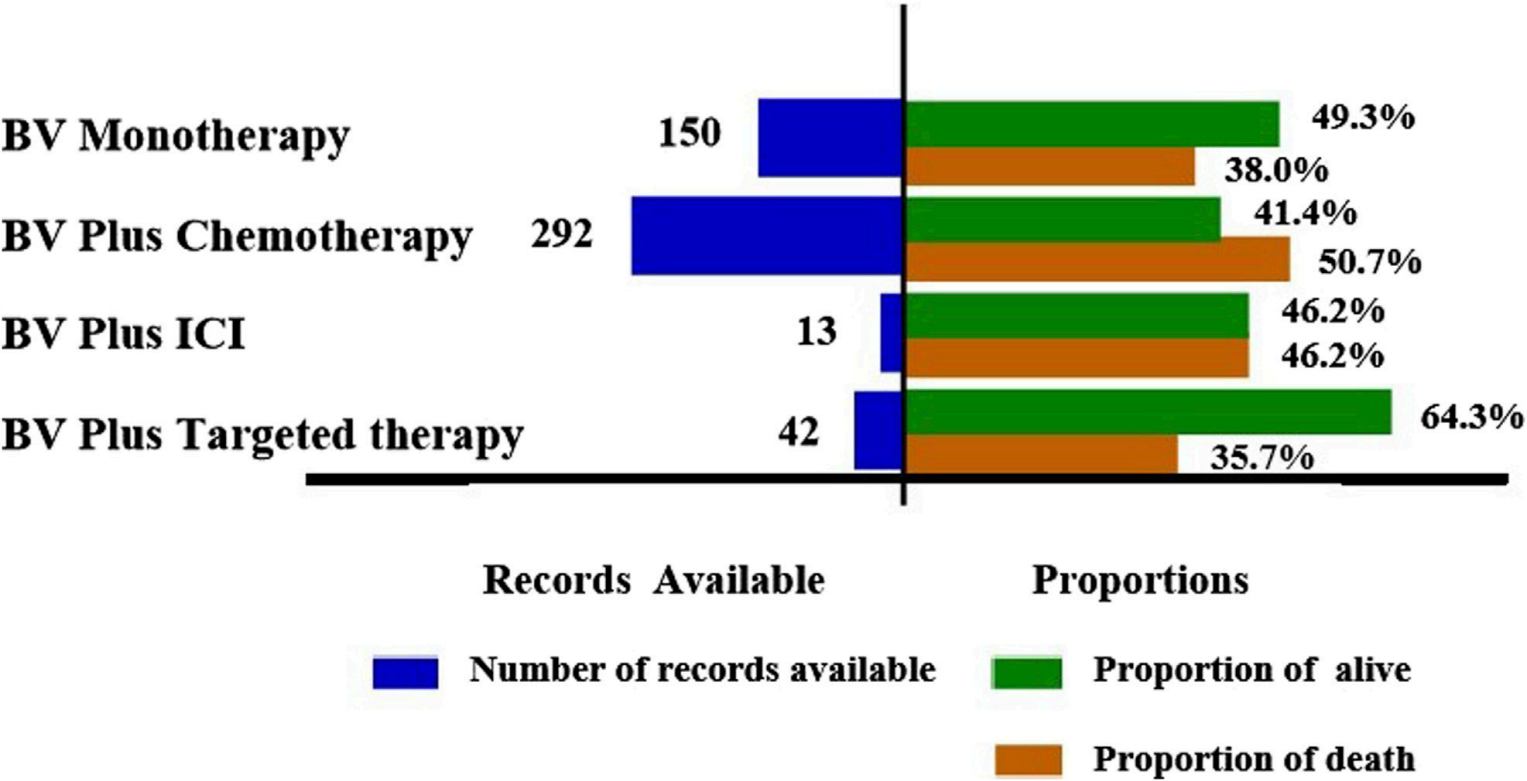
Records and proportions of death and alive in patients with pulmonary haemorrhage and haemoptysis when receiving BV-related regimens.

To explore the prognosis of reports with pulmonary haemorrhage and haemoptysis AEs after the use of various BV-related treatments, our study evaluated the outcome of reports by death and alive proportions. Overall, death accounted for 45.5% of all BV-related pulmonary haemorrhage and haemoptysis AEs records with available outcome information (Table 2). Further subgroup analysis showed the records and proportions of death and alive in patients with pulmonary haemorrhage and haemoptysis when receiving BV-related regimens (Figure 3). As a result, BV plus chemotherapy had the highest percentage of death among the studied cases (148 deaths out of 292 cases, 50.7%), followed by BV plus ICI (6 deaths out of 13 cases, 46.2%), BV monotherapy (57 deaths out of 150 cases, 38.0%), and BV plus targeted therapy had the lowest (15 deaths out of 42 cases, 35.7%). Subsequently, we conducted a comprehensive statistical analysis to describe the clinical characteristics of the death cases, as summarized in Supplementary Table S3. Of the 226 death cases, the proportion of males was higher than females (42.9% vs. 34.6%). Notably, a significant proportion of death cases originated in the United States, accounting for 51.7% (n = 117). Furthermore, among the death cases, the indications for treatment predominantly encompassed lung cancer (59.7%, n = 135), followed by colorectal cancer (11.1%, n = 25), and breast cancer (8.4%, n = 19).

### 3.5 Disproportionality analysis

**FIGURE 4:**
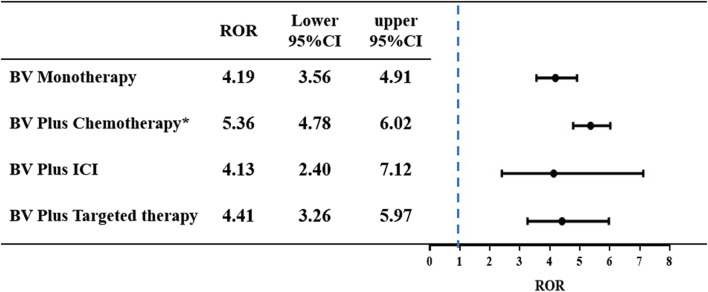
The ROR and 95% CI of pulmonary haemorrhage and haemoptysis cases reported in association with BV-related regimens. *p < 0.05 compared to BV monotherapy group.

The ROR of pulmonary haemorrhage and haemoptysis AEs was calculated for each of the four treatment strategies. The results are shown in Figure 4. A signal of disproportionate reporting was shown when the lower limit of the 95% Cl of ROR exceeded 1, with at least three cases. Among all the treatments, we identified that each of the four BV-related subgroups observed a positive signal of disproportionate reporting (BV monotherapy: ROR 4.19, 95% CI 3.56–4.91; BV plus chemotherapy: ROR 5.36, 95% CI 4.78–6.02; BV plus ICI: ROR 4.13, 95% CI 2.40–7.12; BV plus targeted therapy: ROR 4.41, 95% CI 3.26–5.97). It is noteworthy that there was a significant difference in pulmonary haemorrhage and haemoptysis signals of disproportionate reporting in BV plus chemotherapy as compared with BV monotherapy (ROR 5.35 [95% CI, 4.78–6.02] vs. ROR 4.19 [95% CI, 3.56–4.91], *p* = 0.0147).

## 4 Discussion

As BV is widely used in routine cancer treatment and monotherapy or combination with other agents, it will be especially important to recognize the risks of AEs and intervene promptly to reduce its morbidity and mortality. Of all the common AEs, hemorrhagic events are frequently reported in clinical trials associated with BV (Dotan et al., 2012; Allegra et al., 2013; Bennouna et al., 2013; Cunningham et al., 2013). Among them, BV-induced pulmonary haemorrhage and haemoptysis are rare but the most severe and fatal AEs.

Although the mechanisms of hemorrhage regarding BV have not been clarified, the interaction of BV and VEGF could be one of the possible interpretations. As suggested by Hapani et al., BV might damage vascular integrity by inhibiting endothelial survival and proliferation, particularly in tissues with a high VEGF dependence, such as injured mucosal membrane of the airway or peptic ulcers (Hapani et al., 2010). It is also consistent with our results, lung cancer and colorectal cancer are the two largest proportions of reported indications, accounting for 53.1% and 12.3%, respectively. Haemoptysis and pulmonary haemorrhage were disproportionately reported in patients with lung cancer and colorectal cancer, suggesting the necessity of monitoring haemorrhage in these patients. Moreover, Verheul et al. showed that BV might inhibit the coagulation cascade regulated by tissue factor, whose expression on endothelial cells was induced by VEGF (Verheul and Pinedo, 2007). Consistently, the BV-related treatments including four subgroups in our study demonstrated the positive signals of disproportionate reporting of haemoptysis and pulmonary haemorrhage. Overall, these findings are consistent with those of prior studies.

In this study, cases of pulmonary haemorrhage and haemoptysis identifying BV as a suspect product were reported as having occurred shortly after initiating therapy and often documented death as an outcome, the median onset time was 43 days (IQR 15-117 days) after all BV-related categories initiation, these findings are consistent with previous results in some clinical trials (Hapani et al., 2010; Reck et al., 2012; Amit et al., 2013). Further subgroup analyses showed that the longest median onset time was 90.5 (IQR 34–178.5) days for BV plus targeted therapy, while the shortest of 40.5 (IQR 14–90.25) days for BV plus chemotherapy, and 41 (IQR 25.25–54.5) days for BV plus ICI, 55 (IQR 18–153) days for BV monotherapy, respectively. Clinicians should be alert to the onset of symptoms of pulmonary haemorrhage and haemoptysis immediately from the initial stages of BV-related treatment, especially BV plus chemotherapy and BV plus ICI. On the other hand, although it is not available whether the risk of pulmonary haemorrhage and haemoptysis increased in a dose-dependent manner in our research, continuous monitoring is recommended throughout and beyond the entire treatment period, as some cases of pulmonary haemorrhage and haemoptysis were reported during the long term after the start of administration. Pulmonary haemorrhage and haemoptysis were still observed after more than 360 days in over 15% of cases in both BV plus targeted therapy and monotherapy. In our analyses, death was reported as an outcome in 45.5% of pulmonary hemorrhage and hemoptysis cases, suggesting that clinicians need to pay more attention to preventing pulmonary hemorrhage and hemoptysis, especially the patients with lung cancer or when they are treated with BV plus chemotherapy.

Pulmonary haemorrhage and haemoptysis caused by BV have attracted considerable attention due to its high discontinuation and mortality rates. The increasing application of BV in clinical treatment will undoubtedly result in an increased absolute burden and mortality of pulmonary haemorrhage and haemoptysis. A meta-analysis revealed that BV significantly increased the risk of high-grade pulmonary haemorrhage (RR 3.15; 95% CI 1.15–8.61), among 29 patients with fatal bleeding, pulmonary haemorrhage is most common (67%), followed by central nervous system (CNS) hemorrhage (14%) and GI hemorrhage (12%) (Hapani et al., 2010). Another Japan prospective nested case-control study showed that out of a total of 6,774 patients registered, 23 patients (0.3%) experienced grade ≥3 haemoptysis, of whom 8 (34.8%) recovered, 1 (4.3%) had sequela of impaired consciousness and 14 (60.9%) patients died from haemoptysis (Goto et al., 2016). Although the mortality from BV-related pulmonary haemorrhage and haemoptysis was noted in these previous studies, no further analysis of treatment options was performed. However, when contemplating combination therapy for treatment, it is imperative to thoroughly assess both the clinical benefits and the potential overlapping toxicities of the agents involved. In our study, death was most commonly reported as an outcome among the BV plus chemotherapy subgroup (50.68%), and least commonly reported as an outcome among the BV plus targeted therapy subgroup (35.71%). Disproportionality analysis revealed BV plus chemotherapy (ROR 5.36, 95% CI 4.78–6.02), BV plus targeted therapy (ROR 4.41, 95% CI 3.26–5.97), BV monotherapy (ROR 4.19, 95% CI 3.56–4.91) and BV plus ICI (ROR 4.13, 95% CI 2.40–7.12) are associated with disproportionate reporting of pulmonary haemorrhage and haemoptysis. This might due to the disruption of vascular integrity and the suppression of coagulation cascade by BV. In addition, BV plus chemotherapy group showed a significant higher reporting risk in pulmonary haemorrhage and haemoptysis signals of disproportionate reporting as compared with BV monotherapy (*p* = 0.0147). This result may be attributed to the mechanisms involved. BV might indirectly induce significant damage to the vascular walls infiltrated by cancer cells by enhancing the cytotoxic effect of chemotherapy on tumors (Eskens and Verweij, 2006; Kamba and McDonald, 2007). BV might enhance the thrombocytopenia associated with concurrent chemotherapy, thus promoting hemorrhage (Weltermann et al., 1999). Most chemotherapy agents have hematologic toxicities, such as carboplatin, paclitaxel, 5-fluorouracil and so on. William M. Sikov et al. found that grade ≥3 thrombocytopenia was more common with carboplatin and paclitaxel, which might increase the risk of hemorrhage (Sikov et al., 2015). It should be emphasized that these results still need further studies to confirm, especially BV plus ICI group and BV plus targeted therapy group, their small numbers of records, only 13 and 42 cases, respectively, potentially leading to reporting bias.

Our study has the following limitations: first, Due to the vast amount of information in the FAERS database, some information may be lost (e.g., missing patient demographic information) or duplicated (Bate and Evans, 2009). To reduce the effect, reports were cleaned before analysis. According to the deduplication protocol, the deduplication only eliminated exact duplicate records that were associated with follow-up reports. This means that several probable duplicate records remained in the dataset. So duplicate records and missing information remain a limitation of our study. Database reporting is spontaneous and voluntary, potentially leading to reporting bias and underreporting (Nomura et al., 2015). In the FAERS database, any of the reported events reported by non-healthcare professionals might be associated with limited verification as they might lack standardized clinical confirmation. Second, In terms of signal mining methods, the ROR method itself will bring some inevitable false positive signals. Moreover, the lack of information about the total number of drug-exposed patients is another limitation because it makes impossible to calculate event rates in the absence of denominators. Third, the reporting of the association between BV-related treatments and pulmonary haemorrhage and haemoptysis AEs risk may be influenced by the clinical status of the patient, comorbid conditions and other concomitant drugs (e.g., chemotherapy, ICIs or targeted therapy), those potential confounding factors could lead to pulmonary haemorrhage and haemoptysis AEs. Notably, clinical data are not available (or do not allow to fully assess the role of comorbidities). Fourth, the disproportionality analyses do not inform on actual risk and may be subject to reporting biases. It was unable to infer an exact causal relationship, the disproportionality analysis neither quantified risk nor existed causality, but only provided an estimation of the signal of disproportionate reporting strength, which was only statistically significant (Huang et al., 2020). Therefore, prospective clinical studies are still needed to confirm the causal relationship between them. Despite these limitations, this retrospective pharmacovigilance study investigated the pulmonary haemorrhage and haemoptysis reported in association with BV-related therapies and identified the pulmonary haemorrhage and haemoptysis AEs signals of disproportionate reporting using the ROR based on the FAERS, which could provide valuable evidence for further studies and clinical practice in this field.

## 5 Conclusion

In conclusion, the present study utilizing real-world data from the FAERS database describes and analyses post-market cases of pulmonary haemorrhage and haemoptysis reported with different BV-related treatments. The disproportionality analysis revealed that the four BV-related treatments (BV plus chemotherapy, BV monotherapy, BV plus ICI and BV plus targeted therapy) are associated with disproportionate reporting of pulmonary haemorrhage and haemoptysis, BV plus chemotherapy showed a significant higher reporting risk in comparison to BV monotherapy. Death was most commonly reported as an outcome of pulmonary hemorrhage and hemoptysis cases. Thus, it is advisable to pay more attention to the pulmonary haemorrhage and haemoptysis AEs in clinical practice of BV-related treatments. Further research and clinical validation are essential to deepen our understanding of this complex relationship and inform refined clinical guidelines for the management of patients receiving BV-related treatments.

## Data Availability

Publicly available datasets were analyzed in this study. This data can be found here: https://fis.fda.gov/extensions/FPD-QDE-FAERS/FPD-QDE-FAERS.html.
