# Supplementary material for: Pulmonary haemorrhage and haemoptysis associated with bevacizumab-related treatment regimens: a retrospective, pharmacovigilance study using the FAERS database
Source: Front Pharmacol. 2024 Jun 24;15:1339505. doi: 10.3389/fphar.2024.1339505 (PMC11228312; doi:10.3389/fphar.2024.1339505)
Supplement: Supplementary file 1 [file Table1.DOC]

**Pulmonary haemorrhage and haemoptysis associated with bevacizumab-related treatment regimens: a retrospective, pharmacovigilance study using the FAERS database**

Huiping Hu ^1^, Zhiwen Fu ^1^, Jinmei Liu ^1^, Cong Zhang ^1^, Shijun Li ^1^, Yu Zhang ^1^, Ruxu You ^*, 1, 2^

^1^ *Department of Pharmacy, Union Hospital, Tongji Medical College, Huazhong University of Science and Technology*

^2^ *Department of Pharmacy Administration and Clinical Pharmacy, School of Pharmaceutical Sciences, Peking University*

Table S1 The time to onset of pulmonary haemorrhage and haemoptysis cases reported for BV-related regimens.

Table S2 The median time to onset of pulmonary haemorrhage and haemoptysis cases reported for BV-related regimens.

Table S3. The characteristics of 226 death cases from the FAERS database.

Table S1 The time to onset of pulmonary haemorrhage and haemoptysis cases reported for BV-related regimens.

|  | Time to onset | | | | | |
| --- | --- | --- | --- | --- | --- | --- |
|  | 0-30d | 31-90d | 91-180d | 181-270d | 271-360d | >360d |
| BV Monotherapy, n | 18 | 9 | 6 | 2 | 0 | 8 |
| BV Plus Chemotherapy, n | 67 | 46 | 17 | 9 | 3 | 8 |
| BV Plus ICI, n | 2 | 2 | 0 | 0 | 0 | 0 |
| BV Plus Targeted therapy, n | 4 | 5 | 5 | 1 | 1 | 3 |

Table S2 The median time to onset of pulmonary haemorrhage and haemoptysis cases reported for BV-related regimens.

|  | Time to onset (days) | |
| --- | --- | --- |
|  | Median | Interquartile range(IQR) |
| BV Monotherapy | 55 | 18-153 |
| BV Plus Chemotherapy | 40.5 | 14-90.25 |
| BV Plus ICI | 41 | 25.25-54.5 |
| BV Plus Targeted therapy | 90.5 | 34-178.5 |

Table S3. The characteristics of 226 death cases from the FAERS database.

| **Characteristics of Death cases** | **Overall (n=226)** | **BV Monotherapy**  **(n=57)** | **BV Plus Chemotherapy**  **(n=148)** | **BV Plus ICI**  **(n=6)** | **BV Plus Targeted therapy**  **(n=15)** |
| --- | --- | --- | --- | --- | --- |
| **Gender** |  |  |  |  |  |
| Female | 89 (34.6%) | 16 (28.1%) | 64 (43.2%) | 2 (33.3%) | 6 (40.0%) |
| Male | 108 (42.9%) | 31 (54.4%) | 67 (45.3%) | 4 (66.7%) | 7 (46.7%) |
| Unknown | 29 (22.5%) | 10 (17.5%) | 17 (11.5%) | 0 (0.0%) | 2 (13.3%) |
| **Reporting year** |  |  |  |  |  |
| 2019-2023 | 36 (15.9%) | 8 (14.0%) | 21 (14.2%) | 6 (100.0%) | 1 (6.7%) |
| 2014-2018 | 80 (35.4%) | 26 (45.6%) | 49 (33.1%) | 0 (0.0%) | 5 (33.3%) |
| 2009-2013 | 69 (30.5%) | 12 (21.1%) | 54 (36.5%) | 0 (0.0%) | 3 (20.0%) |
| 2008 and before | 41 (18.1%) | 11 (19.3%) | 24 (16.2%) | 0 (0.0%) | 6 (40.0%) |
| **Indications** |  |  |  |  |  |
| Lung cancer | 135 (59.7%) | 27 (47.4%) | 97 (65.5%) | 5 (83.3%) | 6 (40.0%) |
| Colorectal cancer | 25 (11.1%) | 5 (8.8%) | 19 (12.8%) | 0 (0.0%) | 1 (6.7%) |
| Breast cancer | 19 (8.4%) | 3 (5.3%) | 13 (8.8%) | 0 (0.0%) | 3 (20.0%) |
| Renal cancer | 7 (3.1%) | 7 (12.3%) | 0 (0.0%) | 0 (0.0%) | 0 (0.0%) |
| Gastric cancer | 2 (0.9%) | 0 (0.0%) | 2 (1.4%) | 0 (0.0%) | 0 (0.0%) |
| Head and neck cancer | 2 (0.9%) | 0 (0.0%) | 1 (0.7%) | 0 (0.0%) | 1 (6.7%) |
| Ovarian cancer | 0 (0.0%) | 0 (0.0%) | 0 (0.0%) | 0 (0.0%) | 0 (0.0%) |
| Uterus cancer | 3 (1.3%) | 0 (0.0%) | 3 (2.0%) | 0 (0.0%) | 0 (0.0%) |
| Liver cancer | 2 (0.9%) | 1 (1.8%) | 0 (0.0%) | 1 (16.7%) | 0 (0.0%) |
| Others | 10 (4.4%) | 5 (8.8%) | 3 (2.0%) | 0 (0.0%) | 2 (13.3%) |
| Unspecified | 21 (9.3%) | 9 (15.8%) | 10 (6.8%) | 0 (0.0%) | 2 (13.3%) |
| **Reported countries** |  |  |  |  |  |
| United states | 117 (51.7%) | 39 (68.4%) | 67 (45.3%) | 2 (33.3%) | 9 (60.0%) |
| Japan | 31 (13.7%) | 5 (8.8%) | 24 (16.2%) | 2 (33.3%) | 0 (0.0%) |
| China | 7 (3.0%) | 3 (5.3%) | 3 (2.0%) | 0 (0.0%) | 1 (6.7%) |
| United kingdom | 18 (8.0%) | 2 (3.5%) | 12 (8.1%) | 1 (16.7%) | 3 (20.0%) |
| Germany | 15 (6.6%) | 3 (5.3%) | 12 (8.1%) | 0 (0.0%) | 0 (0.0%) |
| France | 9 (3.9%) | 2 (3.5%) | 7 (4.7%) | 0 (0.0%) | 0 (0.0%) |
| Others | 27 (11.9%) | 3 (5.3%) | 21 (14.2%) | 1 (16.7%) | 2 (13.3%) |
| Unspecified | 2 (0.9%) | 0 (0.0%) | 2 (1.4%) | 0 (0.0%) | 0 (0.0%) |
| **Reporters** |  |  |  |  |  |
| Physicians (MD) | 140 (61.9%) | 32 (56.1%) | 96 (64.9%) | 3 (50.0%) | 9 (60.0%) |
| Pharmacist (PH) | 12 (5.3%) | 5 (8.8%) | 6 (4.1%) | 0 (0.0%) | 1 (6.7%) |
| Consumer (CN) | 13 (5.7%) | 6 (10.5%) | 6 (4.1%) | 1 (16.7%) | 0 (0.0%) |
| health professional (HP) | 10 (4.4%) | 0 (0.0%) | 8 (5.4%) | 2 (33.3%) | 0 (0.0%) |
| Other health professional(OT) | 46 (20.3%) | 13 (22.8%) | 29 (19.6%) | 0 (0.0%) | 4 (26.7%) |
| Unspecified | 5 (2.2%) | 1 (1.8%) | 3 (2.0%) | 0 (0.0%) | 1 (6.7%) |
